# Current and Potential Therapies Targeting Inflammation in NASH

**DOI:** 10.3389/fendo.2021.767314

**Published:** 2021-12-03

**Authors:** Somaya Albhaisi, Mazen Noureddin

**Affiliations:** ^1^Department of Internal Medicine, Virginia Commonwealth University, Richmond, VA, United States; ^2^Karsh Division of Gastroenterology and Hepatology Comprehensive Transplant Center, Cedars Sinai Medical Center, Los Angeles, CA, United States

**Keywords:** non-alcoholic steatohepatitis, inflammation, treatment, macrophages, lymphocytes, cytokines, hepatocellular injury, oxidative stress

## Abstract

Nonalcoholic steatohepatitis (NASH) is the advanced form of nonalcoholic fatty liver disease (NAFLD). It is characterized by hepatic steatosis, inflammation, hepatocellular injury, and fibrosis. Inflammation plays a key role in the progression of NASH and can be provoked by intrahepatic (e.g., lipotoxicity, immune responses, oxidative stress and cell death) and extrahepatic sources (adipose tissue or gut). The identification of triggers of inflammation is central to understanding the mechanisms in NASH development and progression and in designing targeted therapies that can halt or reverse the disease. In this review, we summarize the current and potential therapies targeting inflammation in NASH.

## Pathophysiology With Focus on Inflammatory Pathways

Nonalcoholic fatty liver disease (NAFLD) is a prominent cause of liver-related morbidity and mortality (1). Nonalcoholic steatohepatitis (NASH), the advanced form of NAFL, is illustrated by steatosis, inflammation, hepatocyte injury (e.g., ballooning), with or without fibrosis (2). NASH in as many as one-third of patients progresses to cirrhosis, liver-related complications, end-stage liver disease, and the need for liver transplantation (3–5), hepatocellular carcinoma, and death (6). NASH is the second commonest cause for liver transplantation; it is the leading cause in women, and it is expected to become the leading cause in men as well (7). Further, NASH, especially when advanced, carries a high lifetime financial costs (8).

**Figure 1:**
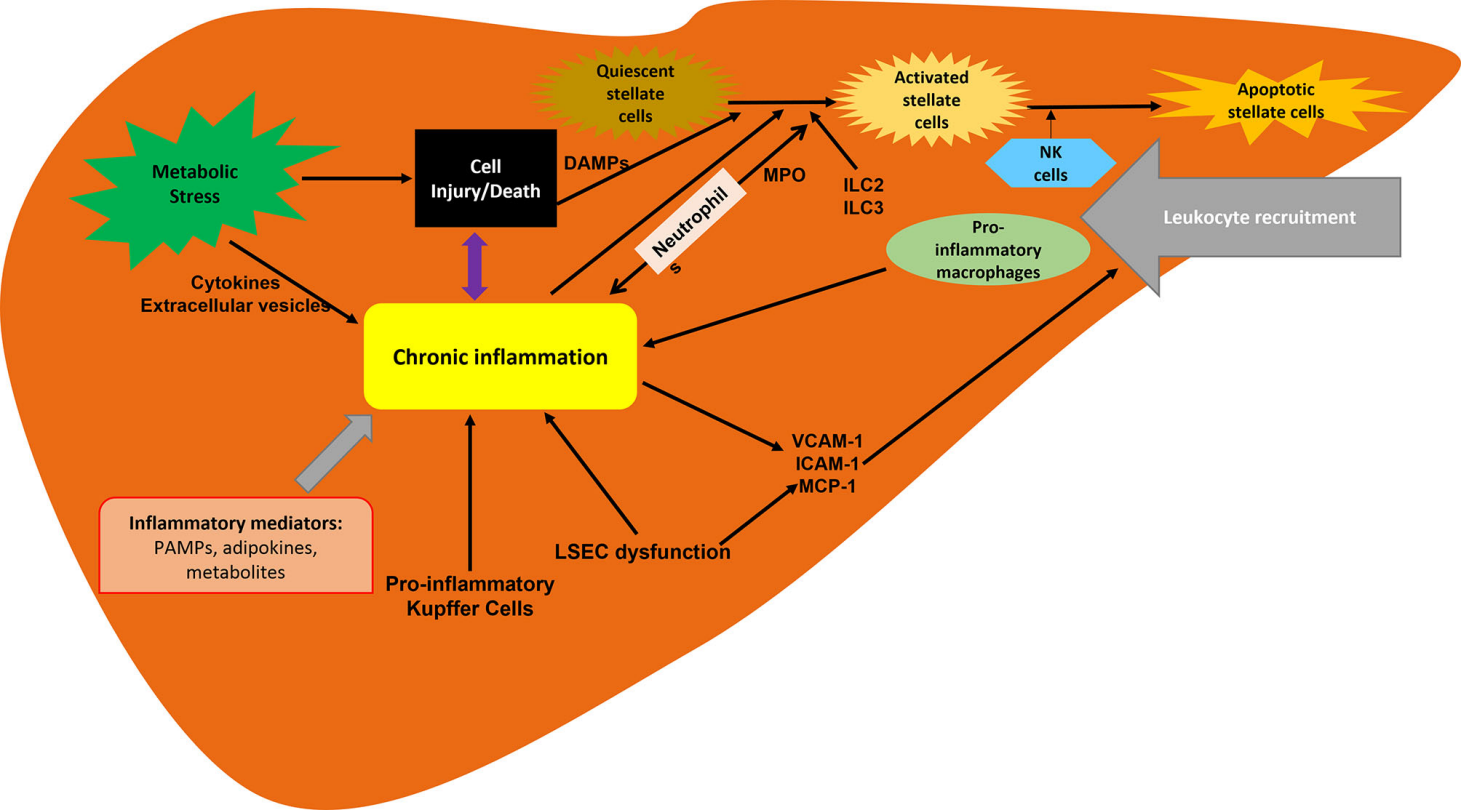
Inflammation associated with NASH. Inflammation is driven by the cellular interplay involving hepatocytes, hepatic stellate cells and recruited immune cells. Hepatocyte death and inflammatory mediators are key contributors to inflammation in NAFLD and NASH progression. ILC, innate lymphoid cells; LSECs, liver sinusoidal endothelial cells; MCP-1, macrophage chemotactic protein 1; MPO, myeloperoxidase.

Much progress has been made in understanding the pathogenesis of NASH and the complex and multifactorial (9, 10) molecular pathways in its development and progression. The hepatic inflammatory response is a key factor in disease progression and the transition from NAFLD to NASH. Inflammation promotes liver fibrosis, which ultimately leads to cirrhosis (11, 12). The presence of histological inflammation has been proposed as an independent predictor of progression of NASH to advanced fibrosis (13). Therefore, inflammation is a key pathophysiological mechanism and a target for therapeutic intervention (**Figure 1**) (14).

Liver inflammation in NAFLD can be triggered by extrahepatic (e.g. adipose tissue and gut) and/or intrahepatic (e.g. lipotoxicity, oxidative stress and cell death) factors (15). These triggers and the diverse pathways promoting and sustaining inflammation determine the disease’s clinical and histological phenotypes (15). Excessive accumulation of free fatty acids (FFA) in the liver from diet and adipose tissue, in addition to lipogenesis in hepatocytes, lead to accumulation of triglycerides in the liver and thus hepatic steatosis. Increased amounts of visceral adipose tissue is associated with adipose tissue insulin resistance and inflammation and results in a disturbed adipokine balance (low adiponectin and high leptin and tumor necrosis factor (TNF) concentrations) (15). Macrophages in adipose tissue secrete chemokines and cytokines that stimulate hepatic inflammation and insulin resistance. Increased liver uptake of FFAs can produce hepatic lipotoxicity and cell death, which accentuate liver inflammation (15). Dietary components, such as fructose, FFAs, and free cholesterol, contribute to a pro-inflammatory milieu in the liver by stimulating lipotoxicity, mitochondrial dysfunction, oxidative and endoplasmic reticulum (ER) stress, and cell death. Dysfunction of the gut barrier can lead to increased bacterial translocation and levels of pathogen-associated or microorganism-associated molecules, which can trigger an inflammatory reaction through activation of inflammatory cells within the liver (15).

Immune cells are important mediators of inflammation and NAFLD progression. NASH is characterized by B cell and T cell infiltration of the liver. Alterations in regulatory T cell and hepatic dendritic cell homeostasis can trigger immune responses that drive the progression of NASH (16). In animal models, lymphocyte responses contribute to sustained hepatic macrophage stimulation and promote natural killer T cell differentiation and survival. It has been found that the inhibition of lymphocyte recruitment improves steatohepatitis and fibrosis (16). The role of immune response in NASH is still evolving.

Hepatocellular death is closely associated with inflammation. The modes of cell death (apoptosis, necrosis, necroptosis and pyroptosis) use distinct mechanisms to trigger death, which contribute to sterile inflammation and perpetuation of the disease (17, 18). Apoptosis is the most widely-studied type of programmed cell death in NASH. It is mediated by caspases and is triggered by either extrinsic pathway that involves membrane receptors or intrinsic pathway that involves intracellular stress (17). Necrosis is an unregulated form of cell death characterized by the production of reactive oxygen species (ROS) and mitochondrial dysfunction which are key pathologic features of NASH and yet, necrosis is a rare histopathological finding in NASH (17). Necroptosis is a recently described form of organized caspase 8-independent cell necrosis that involves the interaction of receptor protein kinases 1 and 3 (RIP1 and RIP3) (17). The potential role of necroptosis in NASH remains controversial. Pyroptosis is a more recently identified pathway of programmed cell death dependent on inflammasome-mediated caspase 1 activation. Further studies are needed to explore its potential role in NASH (17). The process of hepatocyte cell death leads to release of damage-associated molecular patterns (DAMPs), stress signaling molecules that can trigger inflammatory responses and cause tissue damage, thus playing a role in NASH development (17).

The pathogenesis of NASH involves ER stress, which stimulates the unfolded protein response (UPR) in the ER (19). As reported by Szegezdi et al. (19), one of the UPR molecules is the serine/threonine-protein kinase/endoribonuclease inositol-requiring enzyme 1/X-box binding protein 1, which mediates cell apoptosis *via* the TNF receptor-associated factor 2 (TRAF2) and c-Jun N-terminal kinase (JNK) activation. ER stress is sensed by activating transcription factor (ATF6α), inositol-requiring enzyme 1 (IRE1α), and protein kinase RNA-like ER kinase (PERK), which are ER transmembrane proteins that mediate the apoptotic signaling of the UPR. The signaling outcomes of IRE1α activation include complex X-boxbinding protein 1 (XBP)1 activation, regulated IRE1α-dependent decay (RIDD), and TRAF2 mediated signaling. The IRE1α-XBP1 signaling plays a regulatory role in hepatic steatosis, liver injury and inflammation (20). In NASH, protracted activation of the UPR can lead to apoptosis and cell death (19, 21). Toll-like receptors (TLRs), nucleotide-binding oligomerization domain receptors (NOD-like) receptors (NLRs), and recognition signal receptors are part of immune activation and also play a key role in NASH pathogenies (19). The increased intestinal permeability associated with NASH leads to translocation of pathogen-associated molecular patterns (PAMPs) and lipopolysaccharides (LPS) to the liver and subsequently activation of TLRs. Triggering of TLRs results in a pro-inflammatory cytokine cascade, inducing many cytokines, including transforming growth factor-β (TGF-β), interleukin-1 (IL-1β), and TNFα. The activation of TLR2, TLR4, and TLR9 leads to lipid accumulation, activation of stellate cells, and apoptosis of hepatocytes. This apoptosis exacerbates TLRs activation and further release of cytokines (19). TLRs 4 and 9 induce hepatic inflammation and fibrosis *via* inflammasome activation. The actions of TLR4 are mediated by the nuclear factor-kappa B (NF-κB) and mitogen-activated protein kinase (MAPK) cascades (19, 22, 23). NLR activation results in inflammation and apoptosis of hepatocytes through assembly of the caspase 1-containing inflammasome. NOD1 and NOD2 are highly expressed in hepatocytes and have been associated with inflammation (11). The NOD-like receptor protein 3 inflammasome also is activated in NAFLD (19).

Recently, ER stress has been linked to inflammatory responses secondary to lipotoxicity through the release of pro-inflammatory extracellular vesicles (EVs) (24). EVs are non-nucleated, membrane-derived particles secreted by the cells into the extracellular space and include exosomes, microvesicles, and apoptotic vesicles (25, 26). Intercellular and interorgan crosstalk are key drivers of liver injury and inflammation in NASH. Both soluble mediators and circulating EVs have been implicated in hepatocyte-immune cell crosstalk (26, 27). EVs carry cargoes including proteins, lipids, and nucleic acids. Hepatocellular stress caused by lipotoxicity leads to release of EVs from hepatocytes and adipose tissue which trigger inflammation by the activation of monocytes and macrophages, and promote fibrosis and angiogenesis thus accelerating the progression of NAFLD (26).

The Hedgehog pathway is another important pathway for immune response and tissue regeneration that may be involved in hepatic steatosis. Guy et al. (23) have reported that activity of the Hedgehog pathway correlates with the severity of hepatc pathology – ballooning, portal inflammation, and fibrosis. Moreover, lipotoxicity can activate the Hedgehog pathway, thereby stimulating inflammatory responses, hepatocyte differentiation, and activation of stellate cells, leading to fibrosis (19, 28). Mouse models and early-stage clinical trials have revealed that depletion of certain types of immune cells or inhibiting recruitment of inflammatory cells may alleviate fibrosis (17, 25–27). Paradoxically, inflammatory responses are needed for healing and tissue repair, which often are present during the early stages of liver injury (29). Linkage between histologic resolution of steatohepatitis and improvement in fibrosis in NASH has been described (30): Kleiner et al. (31) have reported that the direction and degree of change in histologic features of disease activity correlate with progression or regression of fibrosis, independent of changes in body weight.

## Assessing the Inflammatory Signals of NASH

The search for possible biomarkers for the diagnosis and monitoring of NASH has attracted attention. Among promising candidates is activated plasminogen activator inhibitor 1 (PAI-1), which is a regulator of fibrinolysis that may play a role in liver fibrosis (32). In the NASH Clinical Research Network, PAI-1 was associated with definite NASH in comparison with non-NASH (Odds ratio 1.2, 95% confidence interval 1.08–1.34, *p* < 0.001) (33). PAI-1 values have also been found higher in NASH patients than in patients with NAFL, and PAI-1 independently predicted the severity of NAFLD histology (34). Other biomarkers that have been associated with liver fibrosis are IL‐8, monocyte chemoattractant protein‐1, soluble IL‐1 receptor I, soluble IL‐2 receptor alpha, resistin, and TNFα (29, 32). In a proof-of-concept study, plasma eicosanoid and other polyunsaturated fatty acid metabolites differentiated NAFL from NASH (35) as did 11,12-dihydroxy-eicosatrienoic acid alone. A panel that included 13,14-dihydro-15-keto prostaglandin D_2_ and 20-carboxy arachidonic acid has been proposesd as another eicosanoid candidate biomarker for the noninvasive diagnosis of NASH (35). Others (34) have proposed plasma caspase-generated cytokeratin-18 fragments – markers of liver cell apoptosis – as non-invasive biomarkers for NASH and fibrosis in NAFLD. Although measurement of cytokeratin-18 fragments had a high specificity for NAFLD and fibrosis, it is insufficiently sensitive to be a screening test for staging NASH (36). The inflammatory markers TNF and IL-8 and the hormones adiponectin and fibroblast growth factor 21 have also been evaluated for their ability to identify a circulating signature of NASH. However, according to Albhaisi et al. (37), these non-invasive measurements of fibrosis in NAFLD/NASH could not reliably differentiate between NAFL and NASH or accurately define disease activity or stage.

Metabolomics have shown promise in distinguishing NASH from NAFL (38). Five hundred forty lipids and amino acids in serum samples were assessed in a testing cohort of 467 biopsy proven NAFL/NASH patients that was validated in a separate cohort of 192 patients,. The candidate metabolites algorithm distinguished NAFL from NASH in the validated cohort with an area under the receiver operating characteristic curve of 0.88 while it was 0.79 in the validation cohort (38).

Abnormal serum liver enzymes are usually the first clinical indication of NAFLD, but a large proportion of patients with NAFLD has normal liver enzyme values (39, 40), and the percent of patients with NAFLD that has normal alanine aminotransferase (ALT) or aspartate aminotransferase (AST) levels varies widely (41). In a large cohort of subjects with NAFLD and normal aminotransferase levels enrolled in the NASH Clinical Research Network studies, clinically significant fibrosis, advanced fibrosis, and cirrhosis were prevalent (41). While normal aminotransferase levels do not assure absence of clinically significant NAFLD, they may help predict histologic response in adults with NASH (42).

Among imaging techniques used in the evaluation of NAFLD/NASH, ultrasonography (US) and computed tomography (CT) have low sensitivity and specificity and may underestimate or miss the diagnosis (43). US cannot accurately disclose disease severity. CT is less accurate than US and has the risk of radiation exposure (44). MR spectroscopy (MRS) and MRI-determined proton density fat fraction (MRI-PDFF) are more accuate than US or CT in the the diagnosis of NAFLD (43, 44). MRS is used exclusively in research studies, but it requires highly trained personnel and is not widely available. MRI-PDFF correlated well with MRS in quantifying liver fat in a NASH trial, thus suggesting that it has promise for clinical utility (38, 44). MRI-PDFF images cannot reliably distinguish NASH from NAFL, although a recent meta-analysis found that changes in MRI-PDFF correlated with steatohepatitis changes present on liver biopsy (45). cT1 is a new technology that showed correlation with hostological steatohepatitis and its changes also can predict clinical outcomes in patients with NAFLD/NASH (46). MR elastography, and US elastography are developing techniques that measure liver stiffness. This capability has the potential to detect fibrosis in NAFLD patients, making these techniques promising for use in clinical practice (38, 45). It is worth mentioning that all these imaging techniques do not capture inflammation but rather they offer an assessment of overall disease activity.

## Previous, Current, and Future Treatments

Reducing inflammation is pivotal to halting fibrosis. Therefore, therapeutic agents that target inflammation may attenuate the progression of NAFLD. Even with targeting other mechanisms (e.g. steatosis) this may also affect inflammation indirectly. For instance, peroxisome proliferator-activated receptor agonists and farnesoid X receptor ligands directly reduce steatosis and fibrosis, respectively, but they also improve inflammation indirectly (47, 48). Discussion of drugs that target metabolic pathways but do not directly target inflammation is outside the scope of this review.

### Vitamin E

Because of its antioxidant effects, vitamin E has been studied in NASH (49). Patients treated with vitamin E had improvement in steatosis and inflammation, but fibrosis did not improve (49). Studies on the possible mechanism by which vitamin E reduces steatosis found that it reduces intrahepatic triglycerides by reducing *de novo* lipogenesis *in vitro* and *in vivo* (50). That research also revealed a bi-directional pathway for oxidative stress in which vitamin E exacerbates *de novo* lipogenesis and intrahepatic triglyceride formation, generating a harmful cycle of steatosis and injury. The long-term safety of administration of vitamin E in treatment of NASH patients is unknown.

### Omega-3 Fatty Acids

The therapeutic potential of omega-3 polyunsaturated fatty acids (n-3 PUFA), mainly docosahaexenoic (DHA) and eicosapentaenoic acid (EPA), is becoming appreciated because of their antioxidant and anti-inflammatory properties. Omega-3 fatty acids may reduce oxidative stress, lipotoxicity, and inflammation in patients with NASH, although this possibility is unproven (51). Possible mechanisms of omega-3 fatty acid activity are that of reducing the production of reactive oxygen species and superoxide scavenging (51, 52). n-3 PUFA help regulate lipid metabolism by inhibiting sterol regulatory element-binding protein 1 and activating peroxisome proliferator-activated receptor α (53). In NAFLD, n-3 PUFA may promote FFA oxidation by modulating the production of adipokines (leptin and adiponectin) that control the interaction between adipose tissue, the liver, and muscle (51, 54). n-3 PUFA may also lessen insulin resistance, lipid accumulation, and pro-inflammatory actions. According to Yang et al. (49), n-3 PUFA may be effective in the early stages of NAFLD but not with more severe NAFLD or NASH. Data about the efficacy of omega-3 fatty acids in NAFLD are conflicting (53, 54). Guidelines of the American Association for the Study of Liver Diseases state that “Omega‐3 fatty acids should not be used as a specific treatment of NAFLD or NASH, but they may be considered to treat hypertriglyceridemia in patients with NAFLD” (2).

### Pentoxifylline

PTX is a methylxanthine that suppresses several pro-inflammatory cytokines, including TNF (55). In animal studies, PTX increased hepatic glutathione levels and decreased the production of oxygen radicals in steatohepatitis (56, 57). In human trials, PTX improved steatosis and lobular inflammation and may have impeded the progression of liver fibrosis in NASH (58). In a one-year randomized control trial, PTX improved hepatic inflammatory activity but not fibrosis in NASH patients (59). In a phase 2 trial (NCT00267670), PTX improved transaminases and histology in patients with NASH, but PTX did not reduce transaminases and did not positively affect the metabolic markers that are postulated to contribute to NASH (58, 59). Evidence to support the use of PTX in NASH remains inconclusive.

### Vitamin D

Roth et al. (60) have reported that in a rat model, vitamin D deficiency exacerbated NAFLD through activation of TLRs, insulin resistance, increased hepatic resistin gene expression, and up‐regulation of hepatic inflammatory and oxidative stress genes. In human studies, vitamin D deficiency was associated with an increased risk for NASH development through activation of the mitogen-activated protein kinase and NF-κB pathways (61, 62). In a phase 2 clinical trial, treatment with 2100 IU vitamin D for 48 weeks improved serum ALT levels in patients with hypovitaminosis D and histology-proven NASH (62, 63). Despite the suggestive evidence of a link between vitamin D and NAFLD, the therapeutic value of the vitamin in NASH has not been proven.

### Selonsertib

Selonsertib is an oral inhibitor of apoptosis signal-regulating kinase 1 (ASK1) that was evaluated in phase III trials in patients with NASH and bridging fibrosis (F3, STELLAR-3) or compensated cirrhosis (F4, STELLAR-4) (64). Selonsertib failed to meet the primary end point and was ineffective in reducing fibrosis in patients with advanced fibrosis due to NASH. The researchers concluded that the lack of clinical efficacy was not due to a failure of target inhibition based on *post hoc* analysis (64). Possible explanations for its lack of efficacy include short treatment period, targeting harder to treat groups (cirrhotics rather than F2-F3), and/or lack of efficacy of the drug in humans. One of the lessons learned from the failure of STELLAR 3 and 4 trials is that such trials were designed and conducted without prior placebo-controlled phase IIb studies or preliminary data from a similar population in phase II (65).

### Caspases

Emricasan is a pan-caspase inhibitor that was effective in preclinical studies (66) but did not improve clinical outcomes nor liver histology in NASH patients with fibrosis or NASH-related cirrhosis and severe portal hypertension (67, 68). In contrary, caspase inhibition made fibrosis and ballooning worse in patients with NASH fibrosis. Potential explanations are that caspase inhibition may have directed cells to alternative mechanisms of cell death, resulting in more liver fibrosis and hepatocyte ballooning (ENCORE-PH (NCT02960204) and ENCORE-NF (NCT02686762) trials), or that inhibition of apoptosis leads to dysregulation of the balance between pro- and anti-fibrotic signalling pathways causing a shift toward fibrogenesis (67).

### Chemokine Receptors 2/5 Inhibitors

Hepatic steatosis lead to cellular stress, inflammation and the release of chemokines, including CCL2, from hepatocytes, Kupffer cells, endothelial cells, and stellate cells. Lefore et al. (63) have proposed that cytokine release promotes the infiltration of circulating CCR2+ monocytes, which differentiate into macrophages. These cells activate hepatic stellate cells, promoting collagen deposition and maintaining an inflammatory environment that contriubes to NASH progression (69). The CENTAUR trial (NCT02217475) is a phase IIb study using cenicriviroc a CCR2/CCR5 inhibitor, in subjects with of NASH with a NAFLD activity score (NAS) ≥4 and F1 to F3 stage fibrosis (70). Cenicriviroc did not improve the severity of steatohepatitis, despite the effect on macrophage accumulation in murine models (69). Nevertheless, combining CCR2/5 inhibitors with one or more drugs that act on other metabolic pathways is a reasonable therapeutic rationale.

### Vascular Adhesion Protein-1

Vascular adhesion protein-1 (VAP-1) is a member of the amine oxidase enzyme family (71). Serum levels of VAP-1 are increased in obesity, diabetes, and inflammatory liver disease, and have been correlated with the severity of obesity and NASH. VAP-1 likely contributes to the storage and distribution of lipids in NAFLD, as Shepherd and colleagues have proposed (71). A study that used human and murine model systems demonstrated that elevations in hepatic VAP-1 expression in NASH can contribute to steatosis, metabolic disturbance, and inflammation (71). This observations suggests that targeting the semicarbazide sensitive amine oxidase capacity of VAP-1 would be a useful adjunct to other therapeutic strategies in NAFLD (71). This year, Terns Pharmaceuticals, Inc. announced initiation of the AVIATION Trial (NCT04897594), a multi-center, randomized, double-blind, dose-ranging, placebo-controlled phase 1b clinical trial to evaluate the safety and efficacy of TERN-201, an orally administered, highly selective inhibitor of VAP-1 in patients with NASH (72). The primary goal of the AVIATION Trial is evaluation of the safety and tolerability of TERN-201 when given for 12 weeks in patients with NASH. The clinical trial will be conducted in two parts: Part 1 of the trial has begun with a dose of 10 mg, while Part 2 is anticipated to enroll additional dose cohorts of TERN-201 based on an interim evaluation of the 10 mg cohort. The trial will also investigate the effects of TERN-201 on NASH imaging biomarkers (e.g. cT1) and NASH serum biomarkers (e.g. CK-18) (72).

### S-Adenosylmethionine

S-adenosylmethionine is the principal biological methyl donor in humans (73), and mice deficient in enzymes involved in SAMe metabolism have developed NASH (40, 74). A possible explanation for this effect is that a normal SAMe level is required to establish the proper ratio of phosphatidylethanolamine (PE) to phosphatidylcholine (PC) which plays a role in NAFLD pathogenesis (40). In addition, reduced SAMe levels expose the liver to LPS-induced injury and foster production of pro-inflammatory cytokines (40, 75). SAMe leads to improved liver enzyme values, inflammatory and fibrosis markers, and liver histology in animal models of NASH (38). SAMe has been suggested as an effective therapy in patients with NASH, particularly those who have reduced hepatic SAMe levels (38). Large randomized, placebo-controlled clinical trials to assess the efficacy of SAMe in NASH are needed.

### NLRP3

In a murine model, MCC950, an NLRP3 selective inhibitor, improved NAFLD and fibrosis in obese diabetic mice and reduced liver fibrosis in methionine/choline deficient-fed mice (76). The explanation for this effect maybe blockade of cholesterol crystal-mediated NLRP3 activation in myeloid cells. Therefore, targeting of NLRP3 deserves consideration in the treatment of NASH (76).

### Bromodomain and Extra-Terminal Motif Inhibitors

The BET family of proteins are acetylated histone and non-histone proteins that play an important role in the regulation of pro-inflammatory genes transcription (77). The BET proteins have gained attention due to their potential targets for the treatment of inflammation and other disorders. Several BET inhibitors have been shown to suppress inflammation, reverse fibrosis progression and protect against liver fibrosis in mice models (77, 78). A study in a mouse NASH model to assess the efficacy of a small-molecule BET inhibitor, GSK1210151A (I-BET151), demonstrated reduction of NAS and suppression of interferon-γ expression and fibrosis progression with I-BET151 (78). Inhibition of BET proteins caused alterations in pathways related to lipid metabolism and interferon signaling in addition to reversal of profibrotic gene expression. These results suggest that BET inhibitors may offer a novel therapeutic strategy for the treatment of NASH (78).

### Other Targets

The medium chain free fatty acid receptor G protein-coupled receptor 84 (GPR84) has been suspected of involvement in the development of NAFLD. GPR84 promotes phagocytosis and activation of murine and human macrophages under inflammatory conditions (61, 76). In NAFLD patients, GPR84 expression in the liver was increased, whilst inhibition of GPR84 reduced inflammation and fibrosis in three NASH mouse models (62, 79). Therefore, the potential of GPR84 to treat NAFLD should be assessed.

Among the exceedingly complex relationships of the gut microbiota and human pathophysiology is a possible link to NAFLD. Some of the supporting evidence is amelioration of high-fat-diet-induced NAFLD in mice (80) and reduction of NASH risk in mice on the methionine-choline-deficient diet when treated with antibiotics (81). It is proposed that antibiotics that target the gut-liver axis decrease hepatocellular inflammation (62, 82). In a prospective, phase 2 study (NCT02443116), treatment with aldafermin, an analog of the gut hormone fibroblast growth factor (FGF)19 was associated with a significant, dose‐dependent enrichment in the rare genus *Veillonella*, which correlated with changes in serum bile acid profile and, especially, decreases in toxic bile acids (83). The relationships between microbiome–host interactions and NAFLD deserves further investigation.

## Future Directions

Treatment of NASH is a major unmet need. There are no Food and Drug Administration-approved therapies for the condition. However, interest in combination therapy is growing, consistent with the complexity of the disease and multi-system involvement. As such, adding drugs that directly target inflammation to drugs that have metabolic targets may help expedite strategies to reverse fibrosis and progression of the disease. Hence, there is need for more anti-inflammatory drugs for treatment of NASH, and further studies are needed to improve our understanding of combination therapy. Development of credible non-invasive biomarkers to reliably predict histological and clinical responses to facilitate the selection and monitoring of specific therapies also is needed.

## Author Contributions

All authors listed have made a substantial, direct, and intellectual contribution to the work and approved it for publication.

## Conflict of Interest

MN has been on the advisory board for 89BIO, Gilead, Intercept, Pfizer, Novo Nordisk, Blade, EchoSens, Fractyl, Terns, Siemens and Roche diagnostic; MN has received research support from Allergan, BMS, Gilead, Galmed, Galectin, Genfit, Conatus, Enanta, Madrigal, Novartis, Shire, Viking and Zydus; MN is a minor shareholder or has stocks in Anaetos, Rivus Pharma and Viking.

The remaining author declares that the research was conducted in the absence of any commercial or financial relationships that could be construed as a potential conflict of interest.

## Publisher’s Note

All claims expressed in this article are solely those of the authors and do not necessarily represent those of their affiliated organizations, or those of the publisher, the editors and the reviewers. Any product that may be evaluated in this article, or claim that may be made by its manufacturer, is not guaranteed or endorsed by the publisher.

## References

[1] Younossi ZM Koenig AB Abdelatif D Fazel Y Henry L Wymer M. Global Epidemiology of Nonalcoholic Fatty Liver Disease-Meta-Analytic Assessment of Prevalence, Incidence, and Outcomes. Hepatology (2016) 64(1):73–84. doi: 10.1002/hep.28431 26707365

[2] Chalasani N Younossi Z Lavine JE Charlton M Cusi K Rinella M. The Diagnosis and Management of Nonalcoholic Fatty Liver Disease: Practice Guidance From the American Association for the Study of Liver Diseases. Hepatology (2018) 67(1):328–57. doi: 10.1002/hep.29367 28714183

[3] Marengo A Jouness RIK Bugianesi E. Progression and Natural History of Nonalcoholic Fatty Liver Disease in Adults. Clin Liver Dis (2016) 20(2):313–24. doi: 10.1016/j.cld.2015.10.010 27063271

[4] Goh GB-B McCullough AJ. Natural History of Nonalcoholic Fatty Liver Disease. Dig Dis Sci (2016) 61(5):1226–33. doi: 10.1007/s10620-016-4095-4 PMC7789914 27003142

[5] Matteoni CA Younossi ZM Gramlich T Boparai N Liu YC McCullough AJ. Nonalcoholic Fatty Liver Disease: A Spectrum of Clinical and Pathological Severity. Gastroenterology (1999) 116(6):1413–9. doi: 10.1016/s0016-5085(99)70506-8 10348825

[6] Stine JG Wentworth BJ Zimmet A Rinella ME Loomba R Caldwell SH. Systematic Review With Meta-Analysis: Risk of Hepatocellular Carcinoma in Non-Alcoholic Steatohepatitis Without Cirrhosis Compared to Other Liver Diseases. Aliment Pharmacol Ther (2018) 48(7):696–703. doi: 10.1111/apt.14937 30136293 PMC7495494

[7] Noureddin M Vipani A Bresee C Todo T Kim IK Alkhouri N. NASH Leading Cause of Liver Transplant in Women: Updated Analysis of Indications For Liver Transplant and Ethnic and Gender Variances. Am J Gastroenterol (2018) 113(11):1649–59. doi: 10.1038/s41395-018-0088-6 PMC9083888 29880964

[8] Younossi ZM Tampi R Priyadarshini M Nader F Younossi IM Racila A. Burden of Illness and Economic Model for Patients With Nonalcoholic Steatohepatitis in the United States. Hepatology (2019) 69(2):564–72. doi: 10.1002/hep.30254 30180285

[9] Hardy T Oakley F Anstee QM Day CP. Nonalcoholic Fatty Liver Disease: Pathogenesis and Disease Spectrum. Annu Rev Pathol (2016) 11:451–96. doi: 10.1146/annurev-pathol-012615-044224 26980160

[10] Machado MV Diehl AM. Pathogenesis of Nonalcoholic Steatohepatitis. Gastroenterology (2016) 150(8):1769–77. doi: 10.1053/j.gastro.2016.02.066 PMC4887389 26928243

[11] Koyama Y Brenner DA. Liver Inflammation and Fibrosis. J Clin Invest (2017) 127(1):55–64. doi: 10.1172/JCI88881 28045404 PMC5199698

[12] Lee YA Wallace MC Friedman SL. Pathobiology of Liver Fibrosis: A Translational Success Story. Gut (2015) 64(5):830–41. doi: 10.1136/gutjnl-2014-306842 PMC4477794 25681399

[13] Argo CK Northup PG Al-Osaimi AMS Caldwell SH. Systematic Review of Risk Factors for Fibrosis Progression in Non-Alcoholic Steatohepatitis. J Hepatol (2009) 51(2):371–9. doi: 10.1016/j.jhep.2009.03.019 19501928

[14] Filozof C Chow S-C Dimick-Santos L Chen Y-F Williams RN Goldstein BJ. Clinical Endpoints and Adaptive Clinical Trials in Precirrhotic Nonalcoholic Steatohepatitis: Facilitating Development Approaches for an Emerging Epidemic. Hepatol Commun (2017) 1(7):577–85. doi: 10.1002/hep4.1079 PMC5721443 29404480

[15] Schuster S Cabrera D Arrese M Feldstein AE. Triggering and Resolution of Inflammation in NASH. Nat Rev Gastroenterol Hepatol (2018) 15(6):349–64. doi: 10.1038/s41575-018-0009-6 29740166

[16] Sutti S Albano E. Adaptive Immunity: An Emerging Player in the Progression of NAFLD. Nat Rev Gastroenterol Hepatol (2020) 17(2):81–92. doi: 10.1038/s41575-019-0210-2 31605031 PMC7222953

[17] Wree A Mehal WZ Feldstein AE. Targeting Cell Death and Sterile Inflammation Loop for the Treatment of Nonalcoholic Steatohepatitis. Semin Liver Dis (2016) 36(1):27–36. doi: 10.1055/s-0035-1571272 26870930 PMC4955833

[18] Lambrecht J Tacke F. Controversies and Opportunities in the Use of Inflammatory Markers for Diagnosis or Risk Prediction in Fatty Liver Disease. Front Immunol (2021) 11:634409. doi: 10.3389/fimmu.2020.634409 33633748 PMC7900147

[19] Noureddin M Sanyal AJ. Pathogenesis of NASH: The Impact of Multiple Pathways. Curr Hepatol Rep (2018) 17(4):350–60. doi: 10.1007/s11901-018-0425-7 PMC6677539 31380156

[20] Maiers JL Malhi H. Endoplasmic Reticulum Stress in Metabolic Liver Diseases and Hepatic Fibrosis. Semin Liver Dis (2019) 39(2):235–48. doi: 10.1055/s-0039-1681032 PMC6530577 30912096

[21] Puri P Mirshahi F Cheung O Natarajan R Maher JW Kellum JM. Activation and Dysregulation of the Unfolded Protein Response in Nonalcoholic Fatty Liver Disease. Gastroenterology (2008) 134(2):568–76. doi: 10.1053/j.gastro.2007.10.039 18082745

[22] Jia L Vianna CR Fukuda M Berglund ED Liu C Tao C. Hepatocyte Toll-Like Receptor 4 Regulates Obesity-Induced Inflammation and Insulin Resistance. Nat Commun (2014) 5:3878. doi: 10.1038/ncomms4878 24815961 PMC4080408

[23] Spruss A Kanuri G Wagnerberger S Haub S Bischoff SC Bergheim I. Toll-Like Receptor 4 Is Involved in the Development of Fructose-Induced Hepatic Steatosis in Mice. Hepatology (2009) 50(4):1094–104. doi: 10.1002/hep.23122 19637282

[24] Kakazu E Mauer AS Yin M Malhi H. Hepatocytes Release Ceramide-Enriched Pro-Inflammatory Extracellular Vesicles in an IRE1α-Dependent Manner. J Lipid Res (2016) 57(2):233–45. doi: 10.1194/jlr.M063412 PMC4727419 26621917

[25] Wang H Mehal W Nagy LE Rotman Y. Immunological Mechanisms and Therapeutic Targets of Fatty Liver Diseases. Cell Mol Immunol (2021) 18(1):73–91. doi: 10.1038/s41423-020-00579-3 33268887 PMC7852578

[26] Srinivas AN Suresh D Santhekadur PK Suvarna D Kumar DP. Extracellular Vesicles as Inflammatory Drivers in NAFLD. Front Immunol (2021) 11:627424. doi: 10.3389/fimmu.2020.627424 33603757 PMC7884478

[27] Akers JC Gonda D Kim R Carter BS Chen CC. Biogenesis of Extracellular Vesicles (EV): Exosomes, Microvesicles, Retrovirus-Like Vesicles, and Apoptotic Bodies. J Neurooncol (2013) 113(1):1–11. doi: 10.1007/s11060-013-1084-8 23456661 PMC5533094

[28] Machado MV Diehl AM. The Hedgehog Pathway in Nonalcoholic Fatty Liver Disease. Crit Rev Biochem Mol Biol (2018) 53(3):264–78. doi: 10.1080/10409238.2018.1448752 PMC6033520 29557675

[29] Hossain M Kubes P. Innate Immune Cells Orchestrate the Repair of Sterile Injury in the Liver and Beyond. Eur J Immunol (2019) 49(6):831–41. doi: 10.1002/eji.201847485 31001813

[30] Brunt EM Kleiner DE Wilson LA Sanyal AJ Neuschwander-Tetri BA. Improvements in Histologic Features and Diagnosis Associated With Improvement in Fibrosis in Nonalcoholic Steatohepatitis: Results From the Nonalcoholic Steatohepatitis Clinical Research Network Treatment Trials. Hepatology (2019) 70(2):522–31. doi: 10.1002/hep.30418 PMC6570584 30549292

[31] Miura K Yang L van Rooijen N Ohnishi H Seki E. Hepatic Recruitment of Macrophages Promotes Nonalcoholic Steatohepatitis Through CCR2. Am J Physiol Gastrointest Liver Physiol (2012) 302(11):G1310–21. doi: 10.1152/ajpgi.00365.2011 PMC3378163 22442158

[32] Kaikita K Fogo Agnes B Ma Lijun Schoenhard John A Brown NJ Vaughan Douglas E. Plasminogen Activator Inhibitor-1 Deficiency Prevents Hypertension and Vascular Fibrosis in Response to Long-Term Nitric Oxide Synthase Inhibition. Circulation (2001) 104(7):839–44. doi: 10.1161/hc3301.092803 11502712

[33] Ajmera V Perito ER Bass NM Terrault NA Yates KP Gill R. Novel Plasma Biomarkers Associated With Liver Disease Severity in Adults With Nonalcoholic Fatty Liver Disease. Hepatology (2017) 65(1):65–77. doi: 10.1002/hep.28776 27532276 PMC5191932

[34] Targher G Bertolini L Scala L Zenari L Lippi G Franchini M. Plasma PAI-1 Levels Are Increased in Patients With Nonalcoholic Steatohepatitis. Diabetes Care (2007) 30(5):e31–2. doi: 10.2337/dc07-0109 17468361

[35] Loomba R Quehenberger O Armando A Dennis EA. Polyunsaturated Fatty Acid Metabolites as Novel Lipidomic Biomarkers for Noninvasive Diagnosis of Nonalcoholic Steatohepatitis. J Lipid Res (2015) 56(1):185–92. doi: 10.1194/jlr.P055640 PMC4274066 25404585

[36] Cusi K Chang Z Harrison S Lomonaco R Bril F Orsak B. Limited Value of Plasma Cytokeratin-18 as a Biomarker for NASH and Fibrosis in Patients With Non-Alcoholic Fatty Liver Disease. J Hepatol (2014) 60(1):167–74. doi: 10.1016/j.jhep.2013.07.042 23973932

[37] Long MT Gandhi S Loomba R. Advances in Non-Invasive Biomarkers for the Diagnosis and Monitoring of Non-Alcoholic Fatty Liver Disease. Metabolism (2020) 111:154259. doi: 10.1016/j.metabol.2020.154259 PMC7529729 32387227

[38] Mayo R Crespo J Martínez-Arranz I Banales JM Arias M Mincholé I. Metabolomic-Based Noninvasive Serum Test to Diagnose Nonalcoholic Steatohepatitis: Results From Discovery and Validation Cohorts. Hepatol Commun (2018) 2(7):807–20. doi: 10.1002/hep4.1188 PMC6049064 30027139

[39] Rinella ME Loomba R Caldwell SH Kowdley K Charlton M Tetri B. Controversies in the Diagnosis and Management of NAFLD and NASH. Gastroenterol Hepatol (N Y) (2014) 10(4):219–27. PMC4073533 24976805

[40] Noureddin M Mato JM Lu SC. Nonalcoholic Fatty Liver Disease: Update on Pathogenesis, Diagnosis, Treatment and the Role of S-Adenosylmethionine. Exp Biol Med (Maywood) (2015) 240(6):809–20. doi: 10.1177/1535370215579161 PMC4818965 25873078

[41] Gawrieh S Wilson LA Cummings OW Clark JM Loomba R Hameed B. Histologic Findings of Advanced Fibrosis and Cirrhosis in Patients With NAFLD Who Have Normal Aminotransferase Levels. Am J Gastroenterol (2019) 114(10):1626–35. doi: 10.14309/ajg.0000000000000388 PMC6800246 31517638

[42] Loomba R Sanyal AJ Kowdley KV Terrault N Chalasani NP Abdelmalek MF. Factors Associated With Histologic Response in Adult Patients With Nonalcoholic Steatohepatitis. Gastroenterology (2019) 156(1):88–95.e5. doi: 10.1053/j.gastro.2018.09.021 30222962 PMC6696948

[43] Hernaez R Lazo M Bonekamp S Kamel I Brancati FL Guallar E. Diagnostic Accuracy and Reliability of Ultrasonography for the Detection of Fatty Liver: A Meta-Analysis. Hepatology (2011) 54(3):1082–90. doi: 10.1002/hep.24452 PMC4197002 21618575

[44] Bohte AE van Werven JR Bipat S Stoker J. The Diagnostic Accuracy of US, CT, MRI and 1H-MRS for the Evaluation of Hepatic Steatosis Compared With Liver Biopsy: A Meta-Analysis. Eur Radiol (2011) 21(1):87–97. doi: 10.1007/s00330-010-1905-5 20680289 PMC2995875

[45] Stine JG Munaganuru N Barnard A Wang JL Kaulback K Argo CK. Change in MRI-PDFF and Histologic Response in Patients With Nonalcoholic Steatohepatitis: A Systematic Review and Meta-Analysis. Clin Gastroenterol Hepatol (2020) S1542-3565(20)31220–9. doi: 10.1016/j.cgh.2020.08.061 PMC7914285 32882428

[46] Pavlides M Banerjee R Sellwood J Kelly CJ Robson MD Booth JC. Multiparametric Magnetic Resonance Imaging Predicts Clinical Outcomes in Patients With Chronic Liver Disease. J Hepatol (2016) 64(2):308–15. doi: 10.1016/j.jhep.2015.10.009 PMC4751288 26471505

[47] Jiao Y Lu Y Li X. Farnesoid X Receptor: A Master Regulator of Hepatic Triglyceride and Glucose Homeostasis. Acta Pharmacol Sin (2015) 36(1):44–50. doi: 10.1038/aps.2014.116 25500875 PMC4571315

[48] Choudhary NS Kumar N Duseja A. Peroxisome Proliferator-Activated Receptors and Their Agonists in Nonalcoholic Fatty Liver Disease. J Clin Exp Hepatol (2019) 9(6):731–9. doi: 10.1016/j.jceh.2019.06.004 PMC6926194 31889755

[49] Sanyal AJ Chalasani N Kowdley KV McCullough A Diehl AM Bass NM. Pioglitazone, Vitamin E, or Placebo for Nonalcoholic Steatohepatitis. N Engl J Med (2010) 362(18):1675–85. doi: 10.1056/NEJMoa0907929 PMC2928471 20427778

[50] Podszun MC Alawad AS Lingala S Morris N Huang W-CA Yang S. Vitamin E Treatment in NAFLD Patients Demonstrates That Oxidative Stress Drives Steatosis Through Upregulation of De-Novo Lipogenesis. Redox Biol (2020) 37:101710. doi: 10.1016/j.redox.2020.101710 32920226 PMC7494510

[51] Yang J Fernández-Galilea M Martínez-Fernández L González-Muniesa P Pérez-Chávez A Martínez JA. Oxidative Stress and Non-Alcoholic Fatty Liver Disease: Effects of Omega-3 Fatty Acid Supplementation. Nutrients (2019) 11(4):872. doi: 10.3390/nu11040872 PMC6521137 31003450

[52] Richard D Kefi K Barbe U Bausero P Visioli F. Polyunsaturated Fatty Acids as Antioxidants. Pharmacol Res (2008) 57(6):451–5. doi: 10.1016/j.phrs.2008.05.002 18583147

[53] Pettinelli P Del Pozo T Araya J Rodrigo R Araya AV Smok G. Enhancement in Liver SREBP-1c/PPAR-Alpha Ratio and Steatosis in Obese Patients: Correlations With Insulin Resistance and N-3 Long-Chain Polyunsaturated Fatty Acid Depletion. Biochim Biophys Acta (2009) 1792(11):1080–6. doi: 10.1016/j.bbadis.2009.08.015 19733654

[54] Moreno-Aliaga MJ Lorente-Cebrián S Martínez JA. Regulation of Adipokine Secretion by N-3 Fatty Acids. Proc Nutr Soc (2010) 69(3):324–32. doi: 10.1017/S0029665110001801 20540825

[55] Koppe SWP Sahai A Malladi P Whitington PF Green RM. Pentoxifylline Attenuates Steatohepatitis Induced by the Methionine Choline Deficient Diet. J Hepatol (2004) 41(4):592–8. doi: 10.1016/j.jhep.2004.06.030 15464239

[56] Scorletti E Bhatia L McCormick KG Clough GF Nash K Hodson L. Effects of Purified Eicosapentaenoic and Docosahexaenoic Acids in Nonalcoholic Fatty Liver Disease: Results From the WELCOME* Study. Hepatology (2014) 60(4):1211–21. doi: 10.1002/hep.27289 25043514

[57] Sanyal AJ Abdelmalek MF Suzuki A Cummings OW Chojkier M. No Significant Effects of Ethyl-Eicosapentanoic Acid on Histologic Features of Nonalcoholic Steatohepatitis in a Phase 2 Trial. Gastroenterology (2014) 147(2):377–84.e1. doi: 10.1053/j.gastro.2014.04.046 24818764

[58] Zein CO Yerian LM Gogate P Lopez R Kirwan J Feldstein AE. Pentoxifylline Improves Nonalcoholic Steatohepatitis: A Randomized Placebo-Controlled Trial. Hepatology (2011) 54(5):1610–9. doi: 10.1002/hep.24544 PMC3205292 21748765

[59] Alam S Nazmul Hasan S Mustafa G Alam M Kamal M Ahmad N. Effect of Pentoxifylline on Histological Activity and Fibrosis of Nonalcoholic Steatohepatitis Patients: A One Year Randomized Control Trial. J Transl Int Med (2017) 5(3):155–63. doi: 10.1515/jtim-2017-0021 PMC5655462 29085788

[60] Van Wagner LB Koppe SWP Brunt EM Gottstein J Gardikiotes K Green RM. Pentoxifylline for the Treatment of Non-Alcoholic Steatohepatitis: A Randomized Controlled Trial. Ann Hepatol (2011) 10(3):277–86. doi: 10.1016/S1665-2681(19)31539-X 21677329

[61] Nelson JE Roth CL Wilson L Yates K Aouizerat B Morgan–Stevenson V. Vitamin D Deficiency Is Associated With Increased Risk of Nonalcoholic Steatohepatitis in Adults With Nonalcoholic Fatty Liver Disease: Possible Role for MAPK and NF-Kb? Am J Gastroenterol (2016) 111(6):852–63. doi: 10.1038/ajg.2016.51 PMC5361650 27002799

[62] Smeuninx B Boslem E Febbraio MA. Current and Future Treatments in the Fight Against Non-Alcoholic Fatty Liver Disease. Cancers (Basel) (2020) 12(7):1714. doi: 10.3390/cancers12071714 PMC7407591 32605253

[63] Geier A Eichinger M Stirnimann G Semela D Tay F Seifert B. Treatment of Non-Alcoholic Steatohepatitis Patients With Vitamin D: A Double-Blinded, Randomized, Placebo-Controlled Pilot Study. Scand J Gastroenterol (2018) 53(9):1114–20. doi: 10.1080/00365521.2018.1501091 30270688

[64] Harrison SA Wong VW-S Okanoue T Bzowej N Vuppalanchi R Younes Z. Selonsertib for Patients With Bridging Fibrosis or Compensated Cirrhosis Due to NASH: Results From Randomized Phase III STELLAR Trials. J Hepatol (2020) 73(1):26–39. doi: 10.1016/j.jhep.2020.02.027 32147362

[65] Rinella ME Noureddin M. STELLAR 3 and STELLAR 4: Lessons From the Fall of Icarus. J Hepatol (2020) 73(1):9–11. doi: 10.1016/j.jhep.2020.04.034 32360996 PMC7191297

[66] Barreyro FJ Holod S Finocchietto PV Camino AM Aquino JB Avagnina A. The Pan-Caspase Inhibitor Emricasan (IDN-6556) Decreases Liver Injury and Fibrosis in a Murine Model of Non-Alcoholic Steatohepatitis. Liver Int (2015) 35(3):953–66. doi: 10.1111/liv.12570 24750664

[67] Harrison SA Goodman Z Jabbar A Vemulapalli R Younes ZH Freilich B. A Randomized, Placebo-Controlled Trial of Emricasan in Patients With NASH and F1-F3 Fibrosis. J Hepatol (2020) 72(5):816–27. doi: 10.1016/j.jhep.2019.11.024 31887369

[68] Garcia-Tsao G Bosch J Kayali Z Harrison SA Abdelmalek MF Lawitz E. Randomized Placebo-Controlled Trial of Emricasan for Non-Alcoholic Steatohepatitis-Related Cirrhosis With Severe Portal Hypertension. J Hepatol (2020) 72(5):885–95. doi: 10.1016/j.jhep.2019.12.010 31870950

[69] Lefere S Devisscher L Tacke F. Targeting CCR2/5 in the Treatment of Nonalcoholic Steatohepatitis (NASH) and Fibrosis: Opportunities and Challenges. Expert Opin Invest Drugs (2020) 29(2):89–92. doi: 10.1080/13543784.2020.1718106 31952447

[70] Friedman SL Ratziu V Harrison SA Abdelmalek MF Aithal GP Caballeria J. A Randomized, Placebo-Controlled Trial of Cenicriviroc for Treatment of Nonalcoholic Steatohepatitis With Fibrosis. Hepatology (2018) 67(5):1754–67. doi: 10.1002/hep.29477 PMC5947654 28833331

[71] Shepherd EL Karim S Newsome PN Lalor PF. Inhibition of Vascular Adhesion Protein-1 Modifies Hepatic Steatosis *In Vitro* and *In Vivo*. World J Hepatol (2020) 12(11):931–48. doi: 10.4254/wjh.v12.i11.931 PMC7701969 33312420

[72] Terns Announces Initiation of Patient Dosing in AVIATION Phase 1b NASH Clinical Trial of VAP-1 Inhibitor TERN-201 | Terns Pharmaceuticals, Inc. Available at: https://ir.ternspharma.com/news-releases/news-release-details/terns-announces-initiation-patient-dosing-aviation-phase-1b-nash/ (Accessed August 19, 2021).

[73] Mato JM Martínez-Chantar ML Lu SC. S-Adenosylmethionine Metabolism and Liver Disease. Ann Hepatol (2013) 12(2):183–9. doi: 10.1016/S1665-2681(19)31355-9 PMC4027041 23396728

[74] Lu SC. Mato JM. S-Adenosylmethionine in Liver Health, Injury, and Cancer. Physiol Rev (2012) 92(4):1515–42. doi: 10.1152/physrev.00047.2011 PMC3698976 23073625

[75] Chawla RK Bonkovsky HL Galambos JT. Biochemistry and Pharmacology of S-Adenosyl-L-Methionine and Rationale for Its Use in Liver Disease. Drugs (1990) 40 Suppl 3:98–110. doi: 10.2165/00003495-199000403-00010 2081485

[76] Mridha AR Wree A Robertson AAB Yeh MM Johnson CD Van Rooyen DM. NLRP3 Inflammasome Blockade Reduces Liver Inflammation and Fibrosis in Experimental NASH in Mice. J Hepatol (2017) 66(5):1037–46. doi: 10.1016/j.jhep.2017.01.022 PMC6536116 28167322

[77] Middleton SA Rajpal N Cutler L Mander P Rioja I Prinjha RK. BET Inhibition Improves NASH and Liver Fibrosis. Sci Rep (2018) 8:17257. doi: 10.1038/s41598-018-35653-4 30467325 PMC6250695

[78] Chan CH Fang C Yarilina A Prinjha RK Qiao Y Ivashkiv LB. BET Bromodomain Inhibition Suppresses Transcriptional Responses to Cytokine-Jak-STAT Signaling in a Gene-Specific Manner in Human Monocytes. Eur J Immunol (2015) 45(1):287–97. doi: 10.1002/eji.201444862 PMC4293348 25345375

[79] Puengel T De Vos S Hundertmark J Kohlhepp M Guldiken N Pujuguet P. The Medium-Chain Fatty Acid Receptor GPR84 Mediates Myeloid Cell Infiltration Promoting Steatohepatitis and Fibrosis. J Clin Med (2020) 9(4):1140. doi: 10.3390/jcm9041140 PMC7231190 32316235

[80] Jiang C Xie C Li F Zhang L Nichols RG Krausz KW. Intestinal Farnesoid X Receptor Signaling Promotes Nonalcoholic Fatty Liver Disease. J Clin Invest (2015) 125(1):386–402. doi: 10.1172/JCI76738 25500885 PMC4382255

[81] Henao-Mejia J Elinav E Jin C Hao L Mehal WZ Strowig T. Inflammasome-Mediated Dysbiosis Regulates Progression of NAFLD and Obesity. Nature (2012) 482(7384):179–85. doi: 10.1038/nature10809 PMC3276682 22297845

[82] Yu L-X Schwabe RF. The Gut Microbiome and Liver Cancer: Mechanisms and Clinical Translation. Nat Rev Gastroenterol Hepatol (2017) 14(9):527–39. doi: 10.1038/nrgastro.2017.72 PMC6467288 28676707

[83] Loomba R Ling L Dinh DM DePaoli AM Lieu HD Harrison SA. The Commensal Microbe V Eillonella as a Marker for Response to an FGF19 Analog in NASH. Hepatology (2021) 73(1):126–43. doi: 10.1002/hep.31523 PMC7898628 32794259

